# Prematurity With Extrauterine Growth Restriction Increases the Risk of Higher Levels of Glucose, Low-Grade of Inflammation and Hypertension in Prepubertal Children

**DOI:** 10.3389/fped.2020.00180

**Published:** 2020-04-21

**Authors:** Maria D. Ordóñez-Díaz, Juan L. Pérez-Navero, Katherine Flores-Rojas, Josune Olza-Meneses, Maria C. Muñoz-Villanueva, Concepción M. Aguilera-García, Mercedes Gil-Campos

**Affiliations:** ^1^Department of Paediatrics, Maimónides Biomedical Research Institute, Reina Sofía University Hospital, University of Córdoba, Córdoba, Spain; ^2^Centre for Biomedical Research on Rare Diseases (CIBERER-ISCIII), Madrid, Spain; ^3^Unit of Metabolism and Paediatric Research, Maimónides Biomedical Research Institute, Reina Sofia University Hospital, University of Córdoba, Córdoba, Spain; ^4^Laboratory 123, Department of Biochemistry and Molecular Biology II, Centre of Biomedical Research, Institute of Nutrition and Food Technology, University of Granada, Granada, Spain; ^5^Unit of Methodological Support to Research, Maimónides Biomedical Research Institute, Córdoba, Spain

**Keywords:** prematurity, extrauterine growth restriction, inflammation, cytokines, metabolism, hypertension, programming

## Abstract

**Introduction:** An adipose tissue programming mechanism could be implicated in the extrauterine growth restriction (EUGR) of very preterm infants with morbidity in the cardiometabolic status later in life, as has been reported in intrauterine growth restriction. The aim of this study was to assess whether children with a history of prematurity and EUGR, but also with an adequate growth, showed alterations in the metabolic and inflammatory status.

**Methods:** This was a case–control study. A total of 88 prepubertal children with prematurity antecedents were selected: 38 with EUGR and 50 with an adequate growth pattern (PREM group). They were compared with 123 healthy children born at term. Anthropometry, metabolic parameters, blood pressure (BP), C-reactive protein, hepatocyte growth factor (HGF), interleukin-6 (IL-6), IL-8, monocyte chemotactic protein type 1 (MCP-1), neural growth factor, tumour necrosis factor-alpha (TNF-α) and plasminogen activator inhibitor type-1 were analysed at the prepubertal age.

**Results:** EUGR children exhibited higher BP levels and a higher prevalence of hypertension (46%) compared with both PREM (10%) and control (2.5%) groups. Moreover, there was a positive relationship between BP levels and values for glucose, insulin and HOMA-IR only in children with a EUGR history. The EUGR group showed higher concentrations of most of the cytokines analysed, markedly higher TNF-α, HGF and MCP-1 levels compared with the other two groups.

**Conclusion:** EUGR status leads to cardiometabolic changes and a low-grade inflammatory status in children with a history of prematurity, and that could be related with cardiovascular risk later in life.

## Introduction

The literature over the last several decades has highlighted how prematurity not only affects physical growth (1) and neuro-development (2) during infancy and childhood, but also is a possible risk factor for the development of cardiometabolic complications (3) such as hypertension (4), metabolic syndrome (5), type 1 and type 2 diabetes (6), and cardiovascular disease (CVD) (4, 5). The mechanisms involved in this programming have been studied, and postnatal growth may be impactful on this metabolic dysregulation (7, 8). Extrauterine growth restriction (EUGR) represents the failure of very preterm infants to reach their potential growth. At present, multiple definitions for EUGR have been used, including weight-for-age less than the 10th or 3rd percentile (or weight *Z*-score < −1.28 or < −2, respectively) at 28-days postnatal (9), at 36-weeks' postmenstrual age (PMA) (10) or at hospital discharge (11). Despite the major improvement in the survival of preterm infants (12), neonatal advances have remained insufficient to improve their rate of growth, and EUGR continues to be a frequent complication in neonatal units (1, 13, 14). The growth of very preterm infants during the postconceptional period is likely to be in a similar environment as the condition of intrauterine growth restriction (IUGR), which has been associated with different pathologies such as obesity, diabetes, metabolic syndrome and CVD (3, 15). Therefore, EUGR in preterm infants could also be unfavourable, and similar cardiometabolic outcomes should be expected in this population. In fact, a recent review has highlighted that EUGR in preterm infants associates with poor neurodevelopment and lower later anthropometric measures in childhood, but also alterations in cardiometabolic risk markers (16). The effects of this condition may reinforce the approach that the first 1,000 days (from conception to 2 years) is a critical period for human growth and development (17). However, the metabolic and inflammatory consequences of this early stunted growth have been scarcely studied (18, 19), and it is not clear if these outcomes could depend on the postnatal growth restriction *per se* and/or prematurity (20).

So, children born very prematurely and affected by EUGR, compared with those born prematurely with adecuate growth and with healthy term children, could show different patterns in metabolic and inflammatory biomarkers that could increase later cardiovascular risk. The aim of our study was to evaluate the influence of both prematurity and the EUGR condition on the metabolic and inflammatory status at the prepubertal age.

## Materials and Methods

### Study Participants

The present study is a descriptive, analytical, cross-sectional study. A total of 211 prepubertal children at scholar age were selected and assigned to one of the three groups, all born between 1996 and 2008.

The first group included 38 prepubertal children with a history of prematurity (≤32 weeks' gestational age (GA); with birth weight above the 10th percentile (P10) for GA and postnatal growth restriction, defined as weight < P3 at 36-weeks' PMA, and at discharge from the neonatal unit (EUGR group). Fifty-five participants were initially recruited. Then, 11 children were excluded due to failure to obtain consent, and 6 other candidates were in puberty after physical examination or collection of hormonal data. There were no dropouts. The second group was composed of 50 children with a history of prematurity (≤32 weeks' GA; birth weight above P10 for GA) and adequate growth, defined as weight ≥ P3 at 36-weeks' PMA, and at discharge from the neonatal unit (PREM group). Similarly as the EUGR group, PREM children were selected at prepubertal age. The third group included 123 healthy children born at term with adequate weight and height for GA (38–42 weeks' GA and 2,500–3,500 g at birth), with no relevant antecedents of disease, and free of disease after checking for normal both physical and biochemical evaluation (control group). This group comprised prepubertal children, who were attended to in the hospital for minor disease and required blood analysis, but with normal results in clinical and analytical tests. All the participants were recruited from a single tertiary hospital, which is commonly community benchmark for monitoring and treatment of certain pediatric and neonatal pathologies.

The three groups were selected in accordance with the percentile charts for age, sex and GA at birth developed by Carrascosa et al. (21). At follow-up, charts of Hernández et al. (22) were used. Perinatal data on the EUGR and PREM groups were collected and reviewed from the clinical history, retrospectively. EUGR and PREM infants received the same neonatal care and the nutritional protocol established in the neonatal unit. Parenteral nutrition was similar among preterm infants with and without EUGR, and it included carbohydrates, proteins, amino acids, trace elements, vitamins, and long-chain fatty acids amounts according to their immaturity and clinical evolution. These infants also received similar enteral nutrition—initially trophic and then full-feeding with breast milk or the same formula for premature newborns. Data on the duration of parenteral nutrition, day of initiation of enteral feeding, day with maximum weight loss, maximum weight loss rate, days needed to regain birth weight and days to reach enteral full-feeding were collected. At the prepubertal age, these children were free of any disease related to EUGR and prematurity.

This study was conducted according to the guidelines laid down in the Declaration of Helsinki and was approved by the Institutional Ethical Committee of our hospital. Written informed consent was obtained from all the parents or legal guardians, and verbal consent was obtained from all the children. Verbal consent was witnessed and formally recorded.

### Anthropometry and Blood Pressure Measurements

Weight, length, and head and chest circumference at birth were recorded from the clinical history by a retrospective review. The weight of preterm infants was also assessed at 36-weeks' PMA and at discharge, according to the weight percentile charts for age, sex and GA (21) to classify the preterm groups. Preterm children with the diagnosis of IUGR, defined as fetuses or newborns who had failed to achieve normal weight based on previous growth measurements in the pregnancy, and with an estimated fetal weight that is less than the 10th percentile for gestational age (23), were excluded in this study. To assess for fetal growth, birth weight and head circumference centiles for gestational ages 24–42 weeks from Yudkin et al. (24) were used.

A complete physical exploration was conducted for all the participants at the prepubertal age. Weight and length were measured according to standard protocols with a Health Scale® Ade Rgt-200 stadiometer, with subjects barefoot and in minimal clothing. A delay in weight or height were defined as weight or height ≤P10 at the time of evaluation. A delay in weight-height was defined as both weight and height ≤P10 at the time of evaluation. Body mass index (BMI) was calculated as weight (kg)/ height^2^ (m). Z-scores for weight, length, and BMI were calculated using the standard growth percentile charts for the Spanish population (22). All the participants were assessed at the prepubertal age (Tanner 1), which was confirmed with physical exploration and sexual serum hormone levels (follicle stimulating hormone, luteinising hormone, estradiol, and testosterone) at the age at which the data are presented.

Systolic blood pressure (SBP) and diastolic blood pressure (DBP) were measured with a digital random-zero sphygmomanometer (Dinamap V-100) twice by the same observer. The subjects were resting supine for ≥5 min, and a paediatric cuff was placed around the left arm. Percentiles for SBP and DPB, according to the participant's age and sex, were calculated. Hypertension was defined as blood pressure (BP) levels ≥ percentile 95 (p95) and prehypertension as p90–94 (25).

### Biochemical and Pro-inflammatory Biomarkers

Blood samples were collected in all the groups of children at 09.00 h after a 12-h overnight fast, and at rest while lying down and using an indwelling venous line. All the samples, divided into aliquots, were frozen at −80°C until their analysis.

The general biochemical parameters included serum total cholesterol (TC), high-density lipoprotein cholesterol (HDLc), low-density lipoprotein cholesterol (LDLc) and triglycerides (TG). The markers of carbohydrate metabolism were glucose and insulin. Insulin resistance was calculated by the homeostatic model assessment index (HOMA-IR = insulin (mU/l) × glucose (mmol/l)/22.5). Sex hormones, including follicle stimulating hormone and luteinising hormone, estradiol and testosterone were also analysed. These analyses were carried out using the autoanalyser Architect i2000SR and c16000 (Abbott Diagnostics w, Abbott Laboratories).

Inflammatory biomarkers were measured in plasma. C-reactive protein (CRP) levels were quantified using the autoanalyser Architect c16000 (Abbott Diagnostics®, Abbott Laboratories) by turbidimetric immunoassay with latex particles. Plasma hepatocyte growth factor (HGF), interleukin 6 (IL-6), IL-8, IL 1-β, monocyte chemotactic protein type 1 (MCP-1), neural growth factor (NGF), tumour necrosis factor α (TNF-α) and plasminogen activator inhibitor type 1 (PAI-1) were measured using the cytometre Luminex® X MAP^TM^ Technology (Labscan^TM^ 100) with multiplex technology and LINCOplex assay kits to perform an immunoassay on the surface of polyethylene fluorescent-coded microspheres (26).

### Statistical Analysis

All the possible candidates from the database of our neonatal unit were selected. Assuming a difference of 30% in mean values for the main study variables, an α-error of 0.05, a β-error of 0.1 in a bilateral contrast of hypotheses, and a loss to follow-up of 15–20%, 37 EUGR children, 37 PREM children and 111 control children were estimated (1:1:3) to perform the study. All the results were adjusted to the sex, birth weight and age at the time of evaluation in prepubertal stage.

Descriptive analysis was performed for quantitative variables by the estimation of the median and standard deviation (SD), or median and interquartile range (IQR). Qualitative variables were evaluated by counts and percentages (%). The Shapiro-Wilk test was used to determinate the normality of data distribution, and the homogeneity of variances was estimated by the Levene's test. Categorical variables were compared by the χ2 test. Comparisons of quantitative variables among the three groups were performed by the analysis of variance or the Kruskal–Wallis tests. The Student's *t*-test or Mann–Whitney *U*-test was used for comparisons of quantitative variables between two groups. Spearman's rank correlation coefficients (rho) were calculated to evaluate the relationship between the variables collected. In order to identify the metabolic and inflammatory variables associated with prematurity and the EUGR condition, simple logistic regression analyzes were performed, estimating odds ratios (OR) values and 95% confidence intervals (95% CI). The variables that showed an association with a value of *P* < 0.25 were used for the multiple logistic regression analysis. By the method of backward method selection, the variables with values of *P* ≥ 0.15 for the Wald statistic were eliminated one by one from the model until obtaining the estimate of the adjusted OR. P was significant at <0.05. Data analysis was carried out using the software PASW statistics 18 (IBM SPSS, Inc.).

## Results

The most relevant data on the perinatal stage of preterm children with and without EUGR are shown in Table 1. Although EUGR children showed a lower absolute birth weight than PREM children, birth weight percentiles of these groups were similar and above P10 for GA. In addition, none of these children developed an IUGR, according to the inclusion criteria.

**Table 1:** Perinatal data of children with a history of prematurity and extrauterine growth restriction (EUGR group) and those with prematurity without EUGR (PREM group).

**Perinatal data**	**EUGR group (*N =* 38)**	**PREM group (*N* = 50)**	*p-*value^*^
Gestational age (weeks)	29.50 (25.00, 32.00)	29.00 (25.00, 32.00)	0.645
Birth weight (g)	1100.00 (660.00, 1707.00)	1290.00 (796.00, 1510.00)	0.041
Multiple pregnancy (%)	31.60	42.90	0.543
Prenatal morbidity (%)	32.4	16.7	0.002
Prenatal corticosteroids (%)	81.30	57.90	0.003
Cesarean delivery (%)	25.00	28.00	0.619
Apgar test score at minute 1	5.31 ± 2.79	6.55 ± 1.66	0.101
Apgar test score at minute 5	7.56 ± 2.15	7.66 ± 2.28	0.988
Hyaline membrane disease (%)	42.10	36.00	0.707
Bronchopulmonary displasia (%)	23.70	13.00	0.205
Cerebral haemorrhage (%)	21.10	12.20	0.538
Weight at 36 weeks-post-menstural age (g)	1769.45 ± 149.57	2181.58 ± 213.75	<0.001
Weight at discharge (g)	2475.00 (2245.00, 3200.00)	2455.00 (2230.00, 3895.00)	0.923

*Data are expressed as percentage of subjects, mean values (± standard deviations) or medians (interquartile ranges)*.

* *Statistical significance obtained by Student's t test and Mann–Whitney U-test (continuous variables) or chi-square test (proportions)*.

**Figure 1:**
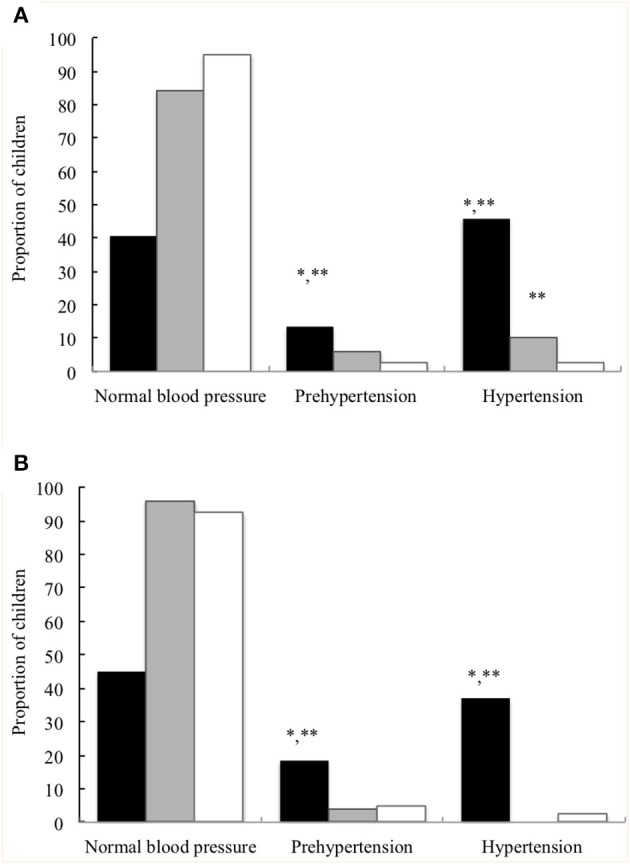
Proportion of children with normal blood pressure percentile, prehypertension, and hypertension at the prepubertal age. **(A)** Systolic blood pressure. **(B)** Diastolic blood pressure. Children with a history of prematurity and extrauterine growth restriction (EUGR group, *N* = 38; ■), children with a history of prematurity without EUGR (PREM group, *N* = 50; ■) and control children (Control group, *N* = 123; □). *Value was significantly different from that of the PREM group with *p*-value < 0.001; **Value was significantly different from that of the control group with *p*-value < 0.001 (Chi-square test).

Demographic and anthropometric parameters, BP and general biochemical markers in all children at the prepubertal age are summarised in Table 2. Significant differences were found in age, sex and Z-scores for BMI at the prepubertal age between the EUGR group and the other two groups. EUGR children showed a higher percentage of weigh-height delays than PREM children, and lower Z-scores for BMI compared with those of the control children. The PREM group exhibited higher values of SBP and a higher prevalence of systolic hypertension than the control group (Figure 1A). However, the EUGR group presented the highest values of BP (Table 2), and the proportion of EUGR children with hypertension and prehypertension was higher in this group (Figures 1A,B).

**Table 2:** Demographic and anthropometric parameters, blood pressure values and biochemical markers in prepubertal children with a history of prematurity and extra uterine growth restriction (EUGR group), those with prematurity without EUGR (PREM group) and healthy children born at term (Control group).

**Prepubertal data**	**EUGR group (*N =* 38)**	**PREM group (*N* = 50)**	**Control group (*N* = 123)**	*p-*value^*^
Age (years)	9.00^a^ (3.00, 13.00)	7.50^b^ (4.00, 12.00)	9.00^a^ (6.00, 12.00)	<0.001
Sex male	27^a^ (71.05)	26^b^ (52.00)	59^b^ (47.97)	<0.050
BMI Z-score	−0.64^a^ (−2.31, 1.75)	−0.41^ab^ (−2.01, 3.52)	−0.19^b^ (−1.18, 0.81)	0.012
Waist Circumference (cm)	57.50^a^ (43.50, 83.00)	59.00^a^ (46.00, 88.00)	58.00^a^ (22.50, 90.00)	0.367
Delay weight-height	5.00 (13.16)^a^	2.00 (4.00)^b^	0.00 (0.00)^c^	<0.001
Delay weight	8 (21.00)^a^	3 (6.00)^b^	0 (0.00)^c^	<0.001
Delay height	9 (24.00)^a^	2 (4.00)^b^	0 (0.00)^c^	<0.001
SBP (mmHg)	114.00^a^ (86.00, 138.00)	101.50^b^ (62.00, 129.00)	90.00^c^ (48.00, 119.00)	<0.001
DBP (mmHg)	72.50^a^ (38.00, 89.00)	58.00^b^ (34.00, 75.00)	59.00^b^ (35.00, 84.00)	<0.001
HDLc (mmol/l)	1.46^a^ (0.32)	1.38^a^ (0.30)	1.72^b^ (0.32)	<0.001
LDLc (mmol/l)	2.43^ab^ (0.54)	2.70^a^ (0.51)	2.37^b^ (0.64)	0.010
TC (mmol/l)	4.21^a^ (0.62)	4.41^a^ (0.61)	4.39^a^ (0.69)	0.314
TG (mmol/l)	0.60^a^ (0.33, 1.60)	0.64^a^ (0.38, 2.29)	0.64^a^ (0.24, 1.21)	0.514
GGT (IU/l)	13.00^a^ (7.00, 29.00)	14.00^a^ (9.00, 24.00)	11.00^b^ (6.00, 23.00)	<0.001
Glucose (mmol/l)	4.91^a^ (3.88, 6.27)	4.66^b^ (3.77, 5.72)	4.61^b^ (3.61, 5.77)	<0.050
Insulin (pmol/l)	32.95^a^ (11.46, 113.17)	47.28^b^ (18.62, 187.67)	35.81^a^ (10.74, 150.42)	<0.050
HOMA-IR	1.02^ab^ (0.29, 3.59)	1.32^a^ (0.50, 5.63)	1.04^b^ (0.26, 5.03)	0.011

*Data are expressed as number (percentage) for sex, weight delay, height delay and weight-height delays; as mean (SD) for HDLc, LDLc and TC; and as median (IQR) for the rest of variables*.

a, b, c *Values within a row with unlike superscript letters were significantly different (p < 0.05), with the P-values expressed*.

* *Statistical significance obtained by analysis of variance, Student's t-test, Kruskal-Wallis and Mann–Whitney U-tests (continuous variables) or chi-square test (proportions)*.

*BMI, body mass index; SBP, systolic blood pressure; DBP, diastolic blood pressure; HDLc, high-density lipoprotein cholesterol; LDLc, low-density lipoprotein cholesterol; TC, total cholesterol; TG, triglycerides; GGT, gamma glutamyl transferase; HOMA-IR, Homeostatic Model Assessment Index; IQR, interquartile range*.

The values of the main biochemical parameters analysed were within the normal range, but it was observed that EUGR and PREM children exhibited lower HDLc and higher GGT values than children in the control group. Higher values of glucose were found in EUGR children compared with the other children. In addition, the PREM group had higher values of LDLc and HOMA-IR than controls, and higher values of insulin than the other two groups (Table 2).

**Figure 2:**
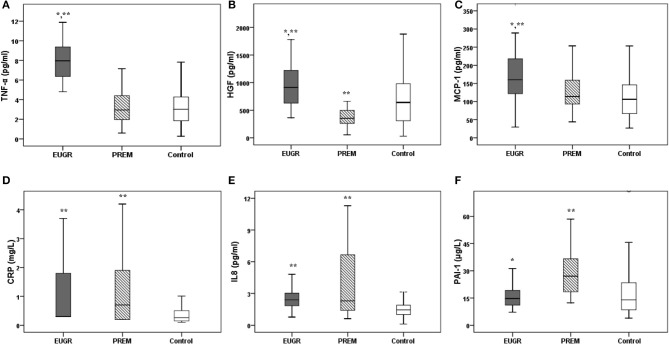
Plasma concentrations of inflammatory biomarkers in prepubertal children with a history of extrauterine growth restriction (EUGR, *N* = 38), prepubertal children with prematurity without EUGR (PREM, *N* = 50) and prepubertal healthy children born at term (Control, *N* = 123). **(A)** TNF-α, tumor necrosis alpha; **(B)** HGF, hepatocyte growth factor; **(C)** MCP-1, monocyte chemotactic protein type 1; **(D)** CRP, C-reactive protein; **(E)** IL-8, interleukin 8; **(F)** PAI-1, plasminogen activator inhibitor type 1; **(G)** IL-6, interleukin 6; **(H)** NGF, neural growth factor. Values are medians, with their interquantile ranges represented by vertical bars. *Value was significantly different from that of the PREM group with *p*-value < 0.005. **Value was significantly different from that of the CONTROL group with *p-*value < 0.005 (Mann– Whitney *U* and Kruskal–Wallis tests).

The values of the most of inflammatory biomarkers are shown in Figures 2A–F. Higher levels of TNF-α, HGF and MCP-1 were observed in the EUGR group compared with the PREM and control groups. Moreover, children in the EUGR and PREM groups showed higher values of CRP and IL-8 than children in the control group. Higher levels of PAI-1 were observed in PREM group compared with the other two groups. No differences were found in IL-6 and NFG values between the three groups:

IL-6: EUGR group 1.64 pg/ml (0.23, 5.81) vs. PREM group 0.95 pg/ml (0.07, 93.31) vs. CONTROL group 1.80 pg/ml (0.02, 55.03), *p* = 0.883

NGF: EUGR group 8.64 pg/ml (3.23, 42.02) vs. PREM group 7.05 pg/ml (1.85, 742.11) vs. CONTROL group 7.69 pg/ml (0.32, 280.25), *p* = 0.697).

The correlation analysis showed that SBP levels had a moderate positive correlation with values of glucose (*rho* = 0.356; *p* = 0.031), insulin (*rho* = 0.349; *p* = 0.034) and HOMA-IR (*rho* = 0.370; *p* = 0.024) only in the EUGR group.

Logistic regression analyses evidenced that an increase in BP levels and a decrease in HDL-c levels at the prepubertal age were statistically associated both prematurity and EUGR conditions (Table 3). Increased TNF-α concentrations and decreased values of Z-score for BMI at the prepubertal age correlated statistically only with prematurity plus a history of EUGR. Values of HGF, IL-8 and PAI-1 were associated with prematurity without EUGR. No significant associations were found between the rest of inflammatory markers and the antecedents of prematurity or EUGR.

**Table 3:** Results of multiple logistic regression analyses to identify the metabolic and inflammatory variables in prepubertal children associated with the conditions of prematurity and extra uterine growth restriction (EUGR group) and prematurity without EUGR (PREM group).

	**EUGR group (*****N*** **= 38)**		**PREM group (*****N*** **= 50)**	
**Parameters**	**Adjusted OR (95% CI)**	***p-*value**	**Adjusted OR (95% CI)**	***p-*value**
Z-score BMI	0.304 (0.131, 0.705)	0.006	0.779 (0.479, 1.265)	0.312
SBP	1.198 (1.121, 1.279)	<0.001	1.075 (1.033, 1.119)	<0.001
HDLc	0.919 (0.874, 0.967)	0.001	0.916 (0.882, 0.950)	<0.001
TNF-α	3.594 (1.411, 9.156)	0.007	^*^	^*^
HGF	0.999 (0.995, 1.002)	0.397	0.994 (0.991, 0.997)	0.001
IL-8	^*^	^*^	3.302 (1.888, 5.777)	<0.001
PAI-1	^*^	^*^	0.824 (0.682, 0.997)	<0.001

*Data are expressed as adjusted odd ratio (OR) values and 95% confidence intervals (CI)*.

*BMI, body mass index; SBP, systolic blood pressure; DBP, diastolic blood pressure; HDLc, high-density lipoprotein cholesterol; TNF-α, tumor necrosis alpha; HGF, hepatocyte growth factor; IL-8, interleukin 8; PAI-1, plasminogen activator inhibitor type 1*.

* *Not applicable. OR value (95% CI) in univariable analysis with p-value > 0.05*.

## Discussion

The present study brings information about the role of prematurity and early postnatal growth restriction on anthropometry, cardio-metabolic profile and inflammatory status of children at the prepubertal age. Our research shows that children with a history of EUGR continue with growth delay during childhood, exhibiting higher BP levels and a disproportionately higher prevalence of hypertension compared with healthy children, but also compared with preterm children without EUGR. BP levels correlated with carbohydrate metabolic parameters only in the EUGR group, and EUGR children showed a higher degree of low-grade inflammation compared with the other two groups. These findings might lead to an increased risk of metabolic diseases and CVD at later ages.

The American Academy of Pediatrics recommends that, under optimum care and appropriate nutritional support, the postnatal growth of preterm infants should approximate the intrauterine growth of foetuses of the same gestational age (27). However, premature birth occurs in neonates at high nutritional risk since many of these infants develop diseases with high caloric requirements, precisely when rapid growth is expected (28). Thus, an EUGR can often be observed in the early postnatal stage (1, 13, 14).

The influence of EUGR on the growth outcomes of preterm children has been highlighted in recent pooled cohorts. Pampanini et al. (29) assessed growth in a cohort of 103 Italian preterm children with severe EUGR, defined as weight and/or length below −2 SD of foetal growth expectations at the time of discharge from hospital, and reported a prevalence of ~13% of subnormal weight and length at 3.9 years. Another study that followed-up 1597 children who were very preterm showed that 7.4% of these children had short stature at 2 years, and 47% of them remained with short stature at 5 years, especially preterm with EUGR (30). Finken et al. (31) reported the follow-up of subjects born prematurely from birth to 19 years of age. Approximately 21% of a total of 380 adolescents evaluated were born with a weight appropriate for gestational age but developed postnatal growth failure and a higher prevalence of short stature (20%). This pattern was similarly observed in preterm infants born small for gestational age and those born appropriate for gestational age with EUGR at 5 years of age. Notably, this height < −2 SD at the age of 5 years pointed to a high risk (≈90%) of short stature in adulthood. Similarly, in a recent research with 411 very-preterm infants (<32 weeks and non-small gestational age), EUGR occurred in 51% at 36–34 postmenstrual weeks, decreasing to 21.1% at 2–2.5 years (1). Some of the variables independently related to the presence of EUGR were lower birth weight and male sex (1). In accordance with all these findings, our EUGR children were predominantly male and showed a lower absolute birth weight (without reaching the 10th percentile) than PREM children (Table 1). Furthermore, preterm children with EUGR showed a higher percentage of weight and height delays compared with preterm children without EUGR (Table 2), and regression analysis showed how the decrease in the BMI Z-score at the prepubertal age was closely associated with the history of EUGR, but not with prematurity without EUGR (Table 3).

Improvements in neonatal care and more aggressive nutritional strategies have aimed to promote postnatal growth (32, 33) and improve neurodevelopment outcomes (33–35). However, these nutritional interventions might also be associated with other adverse health consequences (7). Some reports have highlighted the relationship between preterm birth and the risk of hypertension and CVD (4, 5, 36), but without studying the influence of the EUGR condition. Our findings are consistent with these observations, since we found higher values of SBP in preterm children than in healthy children (Table 2). Mechanisms linking this association are still under debate. Changes in left ventricular geometry, with increased left ventricular mass and significant reductions in systolic and diastolic functional parameters, have been reported in some long-term prospective follow-up studies of preterm-born subjects (37). It has also been hypothesised that a distinct antiangiogenic status with impaired microcirculation (38) or alterations in the organogenesis of kidneys in response to inflammation, oxidative stress, and nephrotoxicity injuries (39, 40) could be involved.

On the other hand, IUGR has also been associated with a higher risk of increased BP values (41) and cardiovascular complications (42). Haemodynamic redistribution and cardiovascular remodelling with an increase arterial thickness in response to adaptation to insufficient nutrition (43) may predict both cardiac dysfunction and hypertension in childhood (44). Interestingly, we observed that preterm children with EUGR showed significantly higher BP levels and a higher proportion of hypertension than the premature group with an adequate rate of growth, and healthy children. Moreover, the percentage of EUGR subjects with BP values between P90-94 was significantly higher than the other children (Figure 1). Thus, a growth failure after premature birth could mimic the unfavourable cardiovascular outcome of the IUGR condition, especially if other harmful factors are associated, such as progressive disturbance of glucose homeostasis. In fact, a higher prevalence of insulin resistance has been described in both preterm and small-for–gestational–age populations (45). In our research, strong positive correlations between BP values and glucose, insulin and HOMA-IR levels were found in EUGR children, but not in the other two groups. Hence, these findings support that an adverse postnatal environment with an EUGR in preterm infants may contribute to the programming of BP and metabolic alterations in childhood.

Some studies have suggested that prematurity might be a risk factor for inflammation activation (39, 46) and the subsequent development of overweight/obesity (47), metabolic syndrome (46) and CVD (39). The influence of IUGR on inflammation and endothelial activation (48, 49), as well as on chronic inflammatory diseases, has also been documented (39). In this line, previous results showed that concentrations of TNF-α, HGF, and MCP-1 were higher in preterm children with EUGR than in healthy children, but these findings were not able to discern whether prematurity or EUGR was involved in this difference (19). In the current study, these cytokines were also higher in the EUGR group compared with the PREM group (Figures 2A–C). Moreover, the multiple regression analysis showed that concentrations of TNF-α in prepubertal children were positively correlated with prematurity only if an EUGR was associated, but not with prematurity and an adequate growth (Table 3). Thus, results evidenced the disadvantage of EUGR after preterm birth to develop a higher inflammatory status. Changes in the adipose tissue and body composition during the early postnatal period, in response to an unfavourable extrauterine environment, could be involved (8). TNF-α, involved markedly in systemic inflammation (50), is chiefly produced by the macrophages of the adipose tissue, and its levels have been positively related to MCP-1 and HGF values (19). MCP-1 is a chemokine that activates monocytes and macrophages toward zones of inflammation implicated in angiogenesis (51), and acts as a proatherosclerotic factor (52). HGF is also secreted by the adipose tissue, and functions as a chemo-attractant for tissue-committed stem cells with potential regeneration, and prompts the secretions of IL-8 and PAI-1 (50).

It has recently been considered that growth failure in preterm infants can be due not only to a limited ability to provide adequate nutritional intake and to acute and chronic illnesses, but also to other factors, such as the inflammation status (33, 35, 53). An inverse relationship between optimal growth and levels of TNF-α, CRP, or IL-6, has been reported (54, 55). The mechanisms regulating the possible negative influence of inflammation on linear growth are not completely known. Some biomarkers, such as growth factors, may be involved. In fact, it has been suggested that higher levels of inflammation could inactivate the growth hormone axis, resulting in elevated growth hormone levels and inappropriately low insulin-like growth factor-1 levels (56). In this study, children with EUGR exhibited a higher weight-height delays (Table 2) and higher values of most of the cytokines analysed (Figure 2) than did the other two groups. Hence, our outcomes might support an interrelation between impaired growth and the proinflammatory status.

There are some limitations in this study that should be noted. EUGR and control groups were older than PREM group, although all the participants were at scholar age and without pubertal signs, according to the inclusion criteria. Body composition, together with anthropometric measurements might influence our results. However, it has been published that recent BMI Z-score is a good indicator of the health status of children, and it is noteworthy that, in our study, it was lower in EUGR and PREM children than in controls. Another possible limitation is the use of national growth tables, although they are commonly used in national studies and were developed with a methodology similar to other international tables such as World Health Organization (WHO) or CDC charts. A moderate concordance has been recently reported between the tables of Hernández et al. and WHO charts (57).

Despite these limitations, the present study has several strengths. On the one hand, this study benefits from a group of children born in the same hospital and with a careful selection of the children, as well as the exhaustiveness of the diagnosis of EUGR and PREM. In previous studies, there is ambiguity regarding the factors involved in the effect of prematurity on long-term health. In addition, we selected school-age children to avoid puberty influence in all the metabolic and anthropometric parameters that can be altered by hormone status. Changes in adipose tissue during normal puberty are known (58). The percentage of visceral fat tends to increase sharply from puberty onwards (58, 59), and associations between body fat depots and cardiometabolic traits have been reported especially after puberty (59). Moreover, it is widely known that insulin resistance appears during puberty (58). In the other hand, some recent research have highlighted that pubertal maturation itself may have an impact on levels of several low-grade inflammation markers (60). For these reasons, associations between biomarkers commonly related with cardiometabolic disease and pro-inflammatory cytokines in both EUGR and prematurity conditions in exclusively prepubertal children were examined. This research is the first to demonstrate that impaired postnatal growth during hospitalisation in very preterm infants could be a particular and independent-determining factor for later metabolic and inflammatory outcomes.

In conclusion, this study highlights that a history of EUGR *per se* increases the risk of growth delay, hypertension and a low-grade of inflammation at the prepubertal age. Based on this study, further prospective, multicentre studies are required to be undertaken, which would be helpful to predict metabolic homeostasis and cardiovascular risk in EUGR populations later in life.

## What Is Known

- There is no consensus on the concept of extrauterine growth restriction (EUGR), defined as weight <10th or 3rd percentile at 36-weeks' postmenstrual age and/or at hospital discharge.
- Prenatal growth can influence metabolic and inflammatory outcomes, and subjects with a history of intrauterine growth restriction show a higher cardiovascular risk.
- EUGR frequently occurs in preterm infants, but its influence in the perinatal programming and later comorbidities has been scarcely studied.

## What It Is New

In our research, strong positive correlations between blood pressure values and glucose, insulin and HOMA-IR levels were found in prepubertal children with a history of EUGR. Values of TNF-α, HGF and MCP-1 were higher in children with a history of prematurity and EUGR than in preterms with an adequate growth pattern and in healthy children. These findings support that an adverse postnatal environment with EUGR in preterm infants may contribute to the programming of blood pressure, to low-grade inflammation and cardiovascular risk later in life.

## Data Availability Statement

All datasets for this study are included in the article/supplementary material.

## Ethics Statement

The studies involving human participants were reviewed and approved by Clinical Research and Bioethics Committee at the Reina Sofia University Hospital, Córdoba (Spain). Written informed consent to participate in this study was provided by the participants' legal guardian/next of kin.

## Author Contributions

MO-D, JP-N, and MG-C contributed to the conception and design of the study, the collection, analysis and interpretation of the data, as well as drafting and revising the manuscript. KF-R, JO-M, and CA-G contributed to the collection, biochemical analysis, and discussion of the data. MM-V performed the statistical analysis. All the authors contributed to the manuscript revision, and read and approved of the version under submission.

## Conflict of Interest

The authors declare that the research was conducted in the absence of any commercial or financial relationships that could be construed as a potential conflict of interest.

## Abbreviations

EUGR: extrauterine growth restriction
PMA: postmenstrual age
IUGR: intrauterine growth restriction
CVD: cardiovascular disease
GA: gestational age
BMI: body mass index
BP: blood pressure
SBP: systolic blood pressure
DBP: diastolic blood pressure
TC: total cholesterol
HDLc: high density lipoprotein cholesterol
LDLc: low density lipoprotein cholesterol
TG: triglycerides
GGT: gamma glutamyl transferase
HOMA-IR: Homeostatic Model Assessment Index
CRP: C-reactive protein
HGF: hepatocyte growth factor
IL: interleukin
MCP-1: monocyte chemotactic protein type 1
NGF: neural growth factor
TNF-α: tumor necrosis factor α
PAI-1: plasminogen activator inhibitor type 1.

## References

[1] Figueras-Aloy J Palet-Trujols C Matas-Barceló I botet-Mussons F Carbonell-Estrany X. Extrauterine growth restriction in very preterm infant: etiology, diagnosis, and 2-year follow-up. Eur J Pediatr. (2020). [Epub ahead of print]. 10.1007/s00431-020-03628-1 32193657

[2] Meyers JM Tan S Bell EF Duncan AF Guillet R Stoll BJ. Eunice kennedy shriver national institute of child health and human development neonatal research network. Neurodevelopmental outcomes among extremely premature infants with linear growth restriction. J Perinatol. (2019) 39:193–202. 10.1038/s41372-018-0259-8 30353080 PMC6351156

[3] Simeoni U Armengaud JB Siddeek B Tolsa JF. Perinatal origins of adult disease. Neonatology. (2018) 113:393–9. 10.1159/00048761 29852488

[4] Bertagnolli M Xie LF Paquette K He Y Cloutier A Oliveira Fernandes R. Endothelial colony-forming cells in young adults born preterm: a novel link between neonatal complications and adult risks for cardiovascular disease. J Am Heart Assoc. (2018) 7:e009720. 10.1161/JAHA.118.009720 29987124 PMC6064846

[5] Markopoulou P Papanikolaou E Analytis A Zoumakis E Siahanidou T. Preterm birth as a risk factor for metabolic syndrome and cardiovascular disease in adult life: a systematic review and meta-analysis. J Pediatr. (2019) 210:69–80.e5. 10.1016/j.jpeds.2019.02.041 30992219

[6] Crump C Sundquist J Sundquist K. Preterm birth and risk of type 1 and type 2 diabetes: a national cohort study. Diabetologia. (2020) 63:508–18. 10.1007/s00125-019-05044-z 31802143 PMC6997251

[7] Ong KK Kennedy K Castañeda-Gutiérrez E Forsyth S Godfrey KM Koletzko B. Postnatal growth in preterm infants and later health outcomes: a systematic review. Acta Paediatr. (2015) 104:974–86. 10.1111/apa.13128 26179961 PMC5054880

[8] Strydom K Van Niekerk E Dhansay MA. Factors affecting body composition in preterm infants: assessment techniques and nutritional interventions. Pediatr Neonatol. (2019) 60:121–8. 10.1016/j.pedneo.2017.10.007 29239827

[9] Makhoul IR Awad E Tamir A Weintraub Z Rotschild A Bader D. Parental and perinatal factors affecting childhood anthropometry of very-low-birth-weight premature infants: a population-based survey. Acta Paediatr. (2009) 98:963–9. 10.1111/j.1651-2227.2009.01242.x 19243350

[10] Lemons JA Bauer CR Oh W Korones SB Papile LA Stoll BJ. Very low birth weight outcomes of the national institute of child health and human development neonatal research network, January 1995 through December 1996. NICHD Neonatal Research Network. Pediatrics. (2001) 107:E1. 10.1542/peds.107.1.e1 11134465

[11] Griffin IJ Tancredi DJ Bertino E Lee HC Profit J. Postnatal growth failure in very low birthweight infants born between 2005 and 2012. Arch Dis Child Fetal Neonatal Ed. (2016) 101:50–5. 10.1136/archdischild-2014-308095 26201534

[12] Patel RM. Short- and long-term outcomes for extremely preterm infants. Am J Perinatol. (2016) 33:318–28. 10.1055/s-0035-1571202 26799967 PMC4760862

[13] Horbar JD Ehrenkranz RA Badger GJ Edwards EM Morrow KA Soll RF. Weight growth velocity and postnatal growth failure in infants 501 to 1500 grams: 2000-2013. Pediatrics. (2015) 136:e84–92. 10.1542/peds.2015-0129 26101360

[14] Avila-Alvarez A Solar Boga A Bermúdez-Hormigo C Fuentes Carballal J. Extrauterine growth restriction among neonates with a birthweight less than 1,500grams. An Pediatr. (2018) 89:325–32. 10.1016/j.anpedi.2018.02.004 29650428

[15] Carolan-Olah M Duarte-Gardea M Lechuga J. A critical review: early life nutrition and prenatal programming for adult disease. J Clin Nurs. (2015) 24:3716–29. 10.1111/jocn.12951 26255862

[16] Martínez-Jiménez MD Gómez-García FJ Gil-Campos M Pérez-Navero JL. Comorbidities in childhood associated with extrauterine growth restriction in preterm infants: a scoping review. Eur J Pediatr. (2020). [Epub ahead of print]. 10.1007/s00431-020-03613-8 32096070

[17] Mameli C Mazzantini S Zuccotti GV. Nutrition in the First 1000 days: the origin of childhood obesity. Int J Environ Res Public Health. (2016) 13:E838. 10.3390/ijerph13090838 27563917 PMC5036671

[18] Ortiz-Espejo M Pérez-Navero JL Olza J Muñoz-Villanueva MC Aguilera CM Gil-Campos M. Changes in plasma adipokines in prepubertal children with a history of extrauterine growth restriction. Nutrition. (2013) 29:1321–5. 10.1016/j.nut.2013.04.015 24012390

[19] Ortiz-Espejo M Pérez-Navero JL Olza-Meneses J Muñoz-Villanueva MC Aguilera-García CM Gil-Campos M. Prepubertal children with a history of extra-uterine growth restriction exhibit low-grade inflammation. Br J Nutr. (2104) 112:338–46. 10.1017/S0007114514000920 24832925

[20] Luu TM Katz SL Leeson P Thébaud B Nuyt AM. Preterm birth: risk factor for early-onset chronic diseases. CMAJ. (2016) 188:736–46. 10.1503/cmaj.150450 26644500 PMC4938684

[21] Carrascosa Lezcano A Ferrández Longás A Yeste Fernández D García-Dihinx Villanova J Romo Montejo A Copil Copil A. Spanish cross-sectional growth study 2008. Part I: weight and height values in newborns of 26-42 weeks of gestational age. An Pediatr (Barc). (2008) 68:544–51. 18559193 10.1157/13123286

[22] Hernández M Castellet J Narvaiza JL Rincón JM Ruiz I Sánchez E Growth Curves and Tables IICD. Madrid: Instituto de investigación sobre crecimiento y desarrollo; Fundación Faustino Orbegozo (1988).

[23] ACOG Practice Bulletin No. 204: Fetal Growth Restriction. Obstet Gynecol. (2019) 133:97–109. 10.1097/AOG.0000000000003070 30681542

[24] Yudkin PL Aboualfa M Eyre JA Redman CWG Wilkinson AR. New birthweight and head circumference centiles for gestational ages 24 to 42 weeks. Early Hum Dev. (1987) 15:45–52. 10.1016/0378-3782(87)90099-5 3816638

[25] National High Blood Pressure Education Program Working Group on High Blood Pressure in Children and Adolescents. The fourth report on the diagnosis, evaluation, and treatment of high blood pressure in children and adolescents. Pediatrics. (2004) 114(2 Suppl.):555–76. 10.1542/peds.2004-2345 15286277

[26] Kellar KL Douglass JP. Multiplexed microsphere-based flow cytometric immunoassays for human cytokines. J Immunol Methods. (2003) 279:277–85. 10.1016/s0301-472x(02)00922-0 12969567

[27] American Academy Paediatrics Committee on Nutrition Nutritional needs of preterm infants. In: Kleinman RE Greer FR editors. Pediatric Nutrition. 7th ed. Elk Grove Village, IL; Washington, DC: American Academy of Pediatrics (2014). p. 83–110.

[28] Fanaro S. Which is the ideal target for preterm growth? Minerva Pediatr. (2010) 62(Suppl. l):77–82. 21089724

[29] Pampanini V Boiani A De Marchis C Giacomozzi C Navas R Agostino R. Preterm infants with severe extrauterine growth retardation (EUGR) are at high risk of growth impairment during childhood. Eur J Pediatr. (2015) 174:33–41. 10.1007/s00431-014-2361-z 24953378

[30] Pierrat V Marchand-Martin L Guemas I Matis J Burguet A Picaud JC. Height at 2 and 5 years of age in children born very preterm: the EPIPAGE study. Arch Dis Child Fetal Neonatal Ed. (2011) 96:348–54. 10.1136/adc.2010.185470 21242241

[31] Finken MJ Dekker FW de Zegher F Wit JM. Dutch project on preterm and small-for-gestational-age-19 collaborative study group. Long-term height gain of prematurely born children with neonatal growth restraint: parallellism with the growth pattern of short children born small for gestational age. Pediatrics. (2006) 118:640–3. 10.1542/peds.2006-0103 16882818

[32] Hiltunen H Löyttyniemi E Isolauri E Rautava S. Early nutrition and growth until the corrected age of 2 years in extremely preterm infants. Neonatology. (2018) 113:100–7. 10.1159/000480633 29131014

[33] Belfort MB Ramel SE. NICU diet, physical growth and nutrient accretion, and preterm infant brain development. Neoreviews. (2019) 20:e385–96. 10.1542/neo.20-7-e385 31261105

[34] Lisa M Hortensius LM van Elburg RM Nijboer CH Benders MJNL de Theije CGM. Postnatal nutrition to improve brain development in the preterm infant: a systematic review from bench to bedside. Front Physiol. (2019) 10:961. 10.3389/fphys.2019.0096 31404162 PMC6677108

[35] Pfister KM Ramel SE. Linear neurodevelopmental outcomes. Clinics in Perinatol. (2014) 41:309–21. 10.1016/j.clp.2014.02.004 24873834

[36] Posod A Odri Komazec I Kager K Pupp Peglow U Griesmaier E Schermer E. Former very preterm infants show an unfavorable cardiovascular risk profile at a preschool age. PLoS ONE. (2016) 11:e0168162. 10.1371/journal.pone.0168162 27959909 PMC5154574

[37] Lewandowski AJ Augustine D Lamata P Davis EF Lazdam M Francis J. Preterm heart in adult life: cardiovascular magnetic resonance reveals distinct differences in left ventricular mass, geometry, and function. Circulation. (2013) 127:197–206. 10.1161/CIRCULATIONAHA.112.126920 23224059

[38] Stritzke A Thomas S Amin H Fusch C Lodha A. Renal consequences of preterm birth. Mol Cell Pediatr. (2017) 4:2. 10.1186/s40348-016-0068-0 28101838 PMC5243236

[39] Nguyen MU Wallace MJ Pepe S Menheniott TR Moss TJ Burgner D. Perinatal inflammation: a common factor in the early origins of cardiovascular disease? Clin Sci. (2015) 129:769–84. 10.1042/CS20150045 26223841

[40] Starzec K Klimek M Grudzien A Jagła M Kwinta P. Longitudinal assessment of renal size and function in extremely low birth weight children at 7 and 11 years of age. Pediatr Nephrol. (2016) 31:2119–26. 10.1007/s00467-016-3413-6 27234909 PMC5039221

[41] Luyckx VA Perico N Somaschini M Manfellotto D Valensise H Cetin I. A developmental approach to the prevention of hypertension and kidney disease: a report from the low birth weight and nephron number working group. Lancet. (2017) 390:424–8. 10.1016/S0140-6736(17)30576-7 28284520 PMC5884413

[42] Menendez-Castro C Rascher W Hartner A. Intrauterine growth restriction - impact on cardiovascular diseases later in life. Mol Cell Pediatr. (2018) 5:4. 10.1186/s40348-018-0082-5 29560535 PMC5861253

[43] Dall'Asta A Brunelli V Prefumo F Frusca T Lees CC. Early onset fetal growth restriction. Matern Health Neonatol Perinatol. (2017) 3:2. 10.1186/s40748-016-0041-x 28116113 PMC5241928

[44] Sehgal A Skilton MR Crispi P. Human fetal growth restriction: a cardiovascular journey through to adolescence. J Dev Orig Health Dis. (2016) 7:626–35. 10.1017/S2040174416000337 27384077

[45] Kopec G Shekhawat PS Mhanna MJ. Prevalence of diabetes and obesity in association with prematurity and growth restriction. Diabetes Metab Syndr Obes. (2017) 10:285–95. 10.2147/DMSO.S115890 28740412 PMC5505541

[46] Nuyt AM Lavoie JC Mohamed I Paquette K Luu TM. Adult consequences of extremely preterm birth: cardiovascular and metabolic diseases risk factors, mechanisms, and prevention avenues. Clin Perinatol. (2017) 44:315–32. 10.1016/j.clp.2017.01.010 28477663

[47] Perrin EM O'Shea TM Skinner AC Bose C Allred EN Fichorova RN. Elevations of inflammatory proteins in neonatal blood are associated with obesity and overweight among 2-year-old children born extremely premature. Pediatr Res. (2018) 83:1110–9. 10.1038/pr.2017.313 29244802 PMC6003823

[48] Pellanda LC Duncan BB Vigo A Rose K Folsom AR Erlinger TP. ARIC Investigators. low birth weight and markers of inflammation and endothelial activation in adulthood: the ARIC study. Int J Cardiol. (2009) 134:371–7. 10.1016/j.ijcard.2008.02.024 18585798 PMC4682734

[49] Lausten-Thomsen U Olsen M Greisen G Schmiegelow K. Inflammatory markers in umbilical cord blood from small-for-gestational-age newborns. Fetal Pediatr Pathol. (2014) 33:114–8. 10.3109/15513815.2013.879239 24476425

[50] Crewe C An YA Scherer PE. The ominous triad of adipose tissue dysfunction: inflammation, fibrosis, and impaired angiogenesis. J Clin Invest. (2017) 127:74–82. 10.1172/JCI88883 28045400 PMC5199684

[51] Salcedo R Ponce ML Young HA Wasserman K Ward JM Kleinman HK. Human endothelial cells express CCR2 and respond to MCP-1: direct role of MCP-1 in angiogenesis and tumor progression. Blood. (2000) 96:34–40. 10.1182/blood.V96.1.34 10891427

[52] Kanda H Tateya S Tamori Y Kotani K Hiasa K Kitazawa R. MCP-1 contributes to macrophage infiltration into adipose tissue, insulin resistance, and hepatic steatosis in obesity. J Clin Invest. (2006) 116:1494–505. 10.1172/JCI26498 16691291 PMC1459069

[53] Ramel SE Demerath EW Gray HL Younge N Boys C Georgieff MK. The relationship of poor linear growth velocity with neonatal illness and two-year neurodevelopment in preterm infants. Neonatology. (2012) 102:19–24. 10.1159/000336127 22441508

[54] Prendergast AJ Rukobo S Chasekwa B Mutasa K Ntozini R Mbuya MN. Stunting is characterized by chronic inflammation in Zimbabwean infants. PLoS ONE. (2014) 9:e86928. 10.1371/journal.pone.0086928 24558364 PMC3928146

[55] Ballinger A. Fundamental mechanisms of growth failure in inflammatory bowel disease. Horm Res. (2002) 58(Suppl. 1):7–10. 10.1159/000064756 12373006

[56] Wong SC Dobie R Altowati MA Werther GA Farquharson C Ahmed SF. Growth and the growth hormone-insulin like growth factor 1 axis in children with chronic inflammation: current evidence, gaps in knowledge, and future directions. Endocrine Rev. (2016) 37:62–110. 10.1210/er.2015-1026 26720129

[57] Aizpurua Galdeano P Mateo Abad M Alonso Á Juaristi Irureta S Carvajal Goikoetxea B García Ruiz S. Effect of changing reference growth charts on the prevalence of short stature. An Pediatr. (2020) 92:28–36. 10.1016/j.anpedi.2019.03.006 31104894

[58] Maffeis C Morandi A. Body composition and insulin resistance in children. Eur J Clin Nutr. (2018) 72:1239–45. 10.1038/s41430-018-0239-2 30185840

[59] Hübers M Geisler C Plachta-Danielzik S Müller MJ. Association between individual fat depots and cardio-metabolic traits in normal- and overweight children, adolescents and adults. Nutr Diabetes. (2017) 7:e267. 10.1038/nutd.2017.20 28481336 PMC5518802

[60] Stumper A Moriarity DP Coe CL Ellman LM Abramson LY Alloy LB. Pubertal status and age are differentially associated with inflammatory biomarkers in female and male adolescents. J Youth Adolesc. (2019). [Epub ahead of print]. 10.1007/s10964-019-01101-3 31410721 PMC7015802

